# CDC20 in and out of mitosis: a prognostic factor and therapeutic target in hematological malignancies

**DOI:** 10.1186/s13046-022-02363-9

**Published:** 2022-04-30

**Authors:** Samantha Bruno, Andrea Ghelli Luserna di Rorà, Roberta Napolitano, Simona Soverini, Giovanni Martinelli, Giorgia Simonetti

**Affiliations:** 1 Department of Experimental, Diagnostic and Specialty Medicine, University of Bologna and Institute of Hematology “L. e A. Seràgnoli”, Bologna, Italy; 2 Biosciences Laboratory, IRCCS Istituto Romagnolo per lo Studio dei Tumori (IRST) “Dino Amadori”, via Piero Maroncelli 40, 47014 Meldola, FC Italy; 3 Scientific Directorate, IRCCS Istituto Romagnolo per lo Studio dei Tumori (IRST) “Dino Amadori”, via Piero Maroncelli 40, 47014 Meldola, FC Italy

**Keywords:** CDC20, Mitotic checkpoint, Apcin, Hematological malignancies

## Abstract

Cell division cycle 20 homologue (CDC20) is a well-known regulator of cell cycle, as it controls the correct segregation of chromosomes during mitosis. Many studies have focused on the biological role of CDC20 in cancer development, as alterations of its functionality have been linked to genomic instability and evidence demonstrated that high CDC20 expression levels are associated with poor overall survival in solid cancers. More recently, novel CDC20 functions have been demonstrated or suggested, including the regulation of apoptosis and stemness properties and a correlation with immune cell infiltration. Here, we here summarize and discuss the role of CDC20 inside and outside mitosis, starting from its network of interacting proteins. In the last years, CDC20 has also attracted more interest in the blood cancer field, being overexpressed and showing an association with prognosis both in myeloid and lymphoid malignancies. Preclinical findings showed that selective CDC20 and APC/C^CDC20^/APC/C^CDH1^ inhibitors, namely Apcin and proTAME, are effective against lymphoma and multiple myeloma cells, resulting in mitotic arrest and apoptosis and synergizing with clinically-relevant drugs. The evidence and hypothesis presented in this review provide the input for further biological and chemical studies aiming to dissect novel potential CDC20 roles and targeting strategies in hematological malignancies.

**Supplementary Information:**

The online version contains supplementary material available at 10.1186/s13046-022-02363-9.

## Background

Cancer hallmarks attempt to organize the complexity of tumor biology into major features. To date, cell growth, signaling regulation, apoptosis evasion, uncontrolled replication, neo-angiogenesis, tissue invasion and metastasis are the most known and investigated mechanisms [1]. An increasing number of studies highlighted the central role of cellular replication, DNA repair, apoptosis and senescence regulation in cancer biology [2]. In eukaryotic cells, cell replication is finely tuned through sequential checkpoint steps that ensure the proper duplication and distribution of the genetic material in daughter cells. Indeed, specialized intracellular pathways control DNA replication, chromosomes condensation and twin chromatids segregation in dividing cells [3, 4] and their dysfunctions can lead to an incomplete or damaged genome, generating cells with a putative oncogenic potential. The relationship between cancer development and deregulation of cellular replication has been well established [5] and is supported by the growing list of genetic or transcriptional alterations affecting key components of the cell cycle regulation machinery in malignant cells [6]. Moreover, cell cycle proteins represent a new class of potential therapeutic targets [7, 8].

Many studies focused on the mechanisms underlying the control of the correct separation of twin chromatids during the final step of cell division. It has been shown that the cell division cycle 20 (CDC20) plays a crucial role in the final steps of mitosis. Recently, there is different evidence which suggest that CDC20 contributes to a number of cellular processes that have been partially explored. An overexpression and/or oncogenic role of *CDC20* has been already described in a variety of human solid tumors [9, 10] including pancreas [11, 12], breast [13, 14], lung [15, 16], prostate [17–19], gastric, colorectal [16, 18], hepatocellular [20, 21], kidney [22], ovarian cancer [23, 24], osteosarcoma [25–27] and glioblastoma [28, 29]. Moreover, *CDC20* expression level has been reported as a putative marker of clinical outcome in many cancer types, being associated with advanced stage, high grade and poor prognosis [9, 10, 30, 31].

The following sections summarize CDC20 functions, including mitosis-related and unrelated ones, and the available data sustaining the oncogenic impact of CDC20 in hematological malignancies in order to evaluate its potential role as prognostic factor and therapeutic target in these neoplasms.

## Main text

### CDC20 role and interactors during the cell cycle

**Fig. 1:**
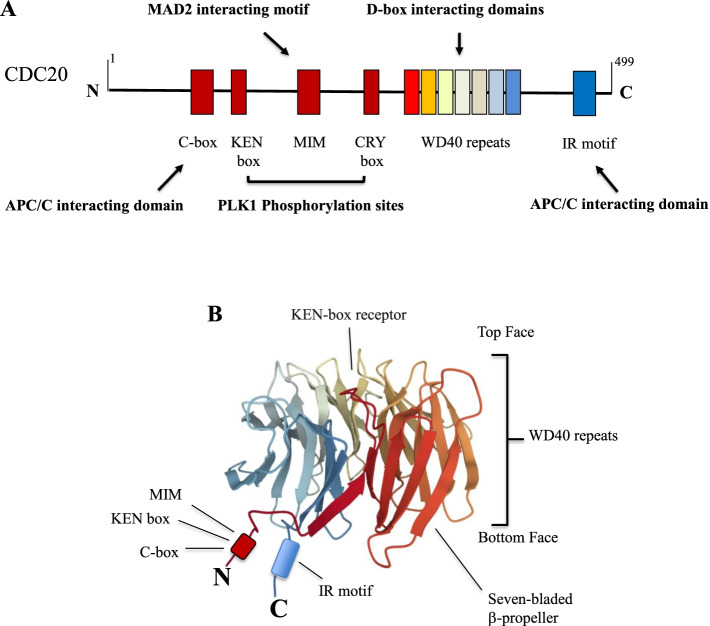
*CDC20* domains and motifs. **A** Structure of human CDC20 with its C-box, KEN box, MAD2-interacting motif (MIM), CRY box, seven WD40 repeats and IR motif. **B** 3D structure of CDC20 (https://www.rcsb.org/)

The *CDC20* gene localizes on the short arm of chromosome 1 and encodes for a 499-amino acid and a 51 kDa protein (Human Protein Atlas proteinatlas.org). CDC20 is composed by two main segments: the N-terminal region characterized by low structural complexity and the C-terminal region containing the WD40 repeats [32]. The N-terminal regions contain different functional structures such as the C-box, KEN-box and CRY-box motifs. The KEN-box and CRY-box represent two independent degradation signals (degrons) and both are crucial binding sites for APC/C. In particular, the CRY-box includes the residue S170, which is phosphorylated by polo-like kinase-1 (PLK1), leading to the timely ubiquitination and destruction of CDC20 [33]. The C-terminal region contains, as reported above, the WD40 repeats and the Ile-Arg (IR) motif. The WD40 repeats define a seven-bladed β-propeller in which two highly conserved surfaces responsible for APC/C degron recognition can be identified: The KEN-box receptor (on the top side) and the D-box co-receptor lying in a channel between blades 1 and 7 of WD40 domain [34]. The KEN-box receptor is crucial for CDC20 regulation. Indeed, two CDC20 regulators, MAD2 and MAD3/BUBR1 interact with CDC20 through different KEN motifs. Additional regulatory regions fall in the amino- and carboxy-terminal extensions and include the C-box, crucial for CDC20’s co-activator function, the MAD2-intercating motif (MIM) and the IR tail, whose function is to bind CDC20 to APC/C. (Fig. 1A-C). Several phosphorylation sites (S41, S42, S72, S92, S153, T157, and S161) relevant for CDC20 functionality have been identified [35]. Mutations of these residues impair checkpoint arrest in mitosis, presumably due to the loss of BUB1-mediated phosphorylation [36].

CDC20 was discovered in 1970 by Hartwell’s group for its role in initiating anaphase and chromosome segregation in yeast models [37]. The key role of CDC20 in mitotic progression has been subsequently demonstrated in mouse models, in which the *CDC20* loss determined embryonic lethality due to prolonged metaphase arrest caused by securin stabilization [38–41], and also in human embryos [42].

**Fig. 2:**
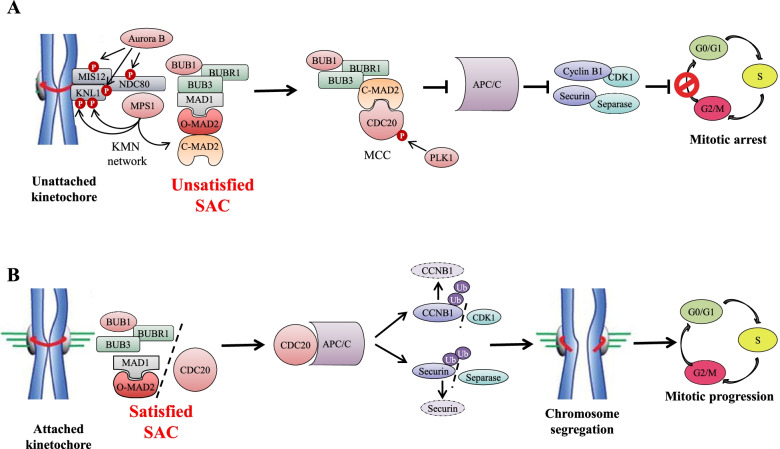
Schematic representation of SAC activity. **A** Unattached or misaligned kinetochores activate the KMN network, leading to recruitment of SAC proteins and conformational change of MAD2 (closed form, C-MAD2). This allows to the formation of the MCC, which sequestrate CDC20 leading to mitotic arrest. **B** Properly attached kinetochores satisfy the SAC, which allow the onset of anaphase through release of CDC20 from MAD2 (open, form, O-MAD2) the interaction between CDC20 and APC/C. APC/C^CDC20^ complex promotes Cyclin B and Securin ubiquitination and degradation, starting sister chromatid separation

During metaphase, CDC20 complexes with MAD1, MAD2, BUBR1, BUB1, and BUB3, to generate the mitotic checkpoint complex (MCC), a crucial effector of the spindle assembly checkpoint (SAC) [43]. The SAC is a multi-protein complex regulating microtubule attachment to each kinetochore during mitosis, in order to avoid the generation of cells with incomplete or altered genomes [44]. Physiologically, the SAC arrests the transition from metaphase to anaphase in the presence of unattached kinetochores, preventing the activation of APC/C [45]. When the kinetochore lacks spindle fibers attachment (SAC complex “unsatisfied”), Aurora B kinase phosphorylates different kinetochore substrates [46, 47] contributing to the recruitment of MPS1 kinase by the KMN network (composed by the 2-subunit KNL1 complex, the 4-subunit MIS12 complex and the 4-subunit NDC80 complex) [48–50]. In turn, MPS1 phosphorylates the kinetochore KNL1 complex at multiple sites [51–53] (Fig. 2A), promoting the localization of the MCC complex on the surface of kinetochores [54]. In detail, phosphorylated KNL1 recruits the BUB3-BUB1 protein complex [55, 56], which is also phosphorylated by MPS1, enabling the interaction with the heterotetrameric MAD1-MAD2 complex [57]. This mechanism promotes the conversion of inactive cytosolic MAD2 into its active conformation [58], a process supported by MPS1 activity [59, 60]. In the nucleus, active MAD2 forms heterodimers with its inactive forms, in order to recruit them to the kinetochore [58]. Lastly, BUB1/BUB3 and MAD2 provide docking sites for BUBR1 and CDC20, which are recruited to the newly formed MCC [61] through spatially and temporally coordinated conformational changes [62]. PLK1 cooperates with the process by phosphorylating CDC20 and keeping it associated with the MCC until all the kinetochores are properly attached to the mitotic spindle fibers (SAC complex “satisfied”, Fig. 2B) [63, 64]. CDC20 is then released from the MCC and binds the APC/C complex. APC/C is a multi-protein complex with a E3-ubiquitin ligase activity that promotes the ubiquitination and proteasomal degradation of several target proteins required for mitotic exit [65, 66], recognized by a D-Box domain [67, 68], a TEK [69] or ABBA motif [61]. APC/C activation and substrate specificity are regulated by the availability of two cofactors, CDC20 and CDH1 [70]. The association and the activity of the APC/C^CDC20^ complex is finely regulated. In addition to CDK1 and other mitotic kinases [71, 72], Tank Binding Kinase 1 (TBK1) [73], CCNB3 [74], Apc1 loop domain (Apc1-loop^500^) [75] and Hematopoietic PBX-interacting protein (HPIP) [76] were also shown to be involved. APC/C^CDC20^ exerts its function during the metaphase to anaphase transition ensuring proper chromatids segregation [35] (Fig. 2B).

The key role exerted by CDC20 during mitosis is further supported by the analysis of the CDC20 interactome. Overall, 817 physical interactions with 171 partners have been reported in humans by proteomic analyses, according to The Biological General Repository for Interaction Datasets (BioGRID, https://thebiogrid.org [77], Table 1). The list of CDC20 interactors is enriched for genes involved in different cell cycle phases, from G1/S to G2/M transition and from DNA damage response to anaphase-promoting complex-dependent catabolic process (Table S1, adjusted *p* < 0.001, KEGG 2021 and Gene Ontology Biological processes pathways 2021, https://amp.pharm.mssm.edu/Enrichr/ [78]). The expression levels of some interactors (*BUB1*, *CCNA2*, *CCNB1*, *CDK1*, *MAD2L1*, and *PLK1*) were positively correlated with *CDC20* in cancer cells [10]. Moreover, the list of interactors included some APC/C^CDC20^ substrates, that are also critical cell cycle regulators, as Securin (PTTG1) [79], CCNB1 [80], CCNA1/2 [81, 82], NEK2 [83, 84], Zwint-1 [85] and p21 [86], indicating an APC/C-mediated role of CDC20 in cell cycle progression and chromosome segregation.

**Table 1:** List of CDC20 interactors identified in human cells

Protein name			
ANAPC1	CDR2	HSPA8	RASSF1
ANAPC10	CDT1	HSPA9	RAVER1
ANAPC11	CEP128	HSPD1	RNF34
ANAPC13	CHFR	HUWE1	RNF4
ANAPC15	CILP	ID1	RNH1
ANAPC16	CKS1B	IST1	RPL23
ANAPC2	CKS2	KDM5B	RPS3
ANAPC4	CLSPN	KIF18A	SIRT2
ANAPC5	COPS5	KRT31	SKP2
ANAPC7	CPVL	KRT32	SPATC1
APP	CREBBP	KRT33A	SPINT2
AURKA	CUEDC2	KRT85	SPOP
AURKB	CUL1	KRTAP19–5	SSSCA1
AXIN2	CUL3	KRTAP9–2	STAU1
BANP	DAXX	KRTAP9–3	TAS2R13
BTRC	DCPS	LNP1	TCP1
BUB1	DSC1	MAD1L1	TFDP1
BUB1B	E2F1	MAD2L1	TGFB1
BUB3	EGLN3	MAD2L1BP	TK1
CAPNS1	EIF2A	MAD2L2	TNIP2
CASC5	EP300	MDC1	TP63
CCDC59	FBXO31	MOV10	TP73
CCNA1	FBXO43	MXI1	TRIM33
CCNA2	FBXO5	MYC	TRIP13
CCNB1	FBXW5	NEK2	TRRAP
CCNF	FGFR1	NINL	TTK
CCT2	FRY	NUP98	TUBA1A
CCT3	FZR1	OTUD7B	TUBA1C
CCT4	GM9174	PARK2	TUBA4A
CCT5	GMNN	PAXIP1	TUBB
CCT6A	HAUS1	PBRM1	TUBB2A
CCT7	HDAC1	PCDHAC2	TUBB4B
CCT8	HDAC2	PCNA	TUBG1
CDC16	HDAC6	PHF8	UBB
CDC23	HECW2	PIM1	UBC
CDC25A	HHEX	PLEKHA4	UBE2C
CDC25B	HIF1AN	PLEKHO2	UBE2S
CDC26	HNRNPF	PLK1	UBR5
CDC27	HOXD13	PPP2CA	USP22
CDC5L	HSF1	PPP2CB	USP37
CDC6	HSF2	PPP2R1A	XPO1
CDK1	HSPA1A	PPP2R5A	YAP1
CDK2	HSPA1L	PPP2R5E	YARS2
CDKN1A	HSPA5	PTTG1	YBX1

### CDC20 functions beyond chromosome segregation

**Fig. 3:**
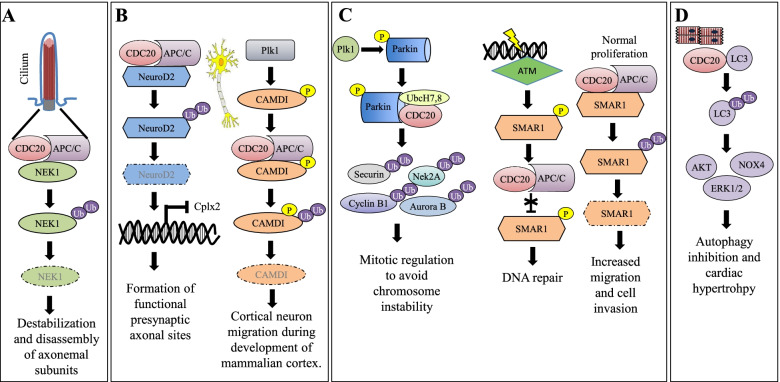
Schematic representation of the role of CDC20 in cell type-specific biological pathways. **A** Ciliary disassembly. **B** Brain development. **C** Genomic stability and DNA repair. **D** Autophagy regulation

**Fig. 4:**
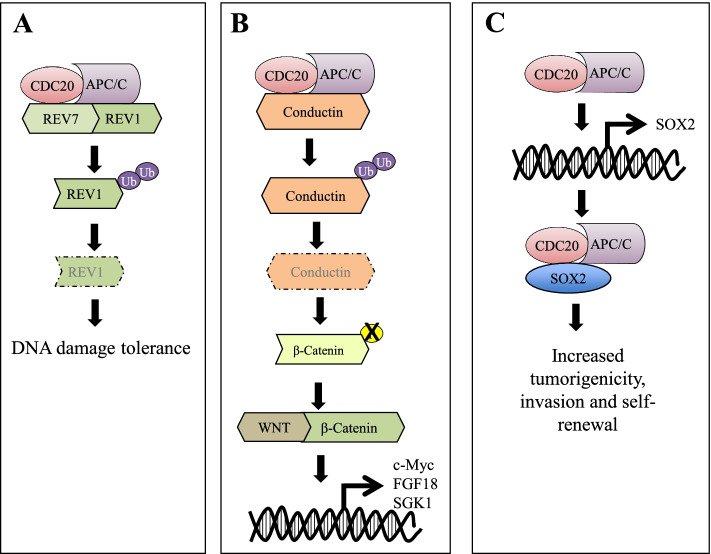
Schematic representation of the role of CDC20 in tumor-associated pathways. **A** Regulation and tolerance of DNA damages. **B** Activation of Wnt/β-catenin signaling. **C** Induction of cell migration and self-renewal

Pathway analysis of CDC20 interacting proteins suggested additional roles of CDC20 outside cell cycle regulation, with a significant enrichment of specific cellular processes, including protein modification, localization and degradation, telomeres regulation, transcription, and other signaling pathways, as Hippo, TGF-β, β-catenin, MAPK (Table S1). Accordingly, it has been recently shown that CDC20 exerts a pivotal role in different cell type-specific biological processes, as ciliary disassembly [87, 88], brain development [89, 90], necrosis suppression in neural stem cells under catastrophic cellular stresses [91], tissue homeostasis and cell fate in human keratinocytes [92, 93], genomic stability [94, 95], aging [96] and autophagy [97, 98] (Fig. 3A-D). Moreover, CDC20 has been associated with cellular processes relevant to tumorigenesis, including the regulation of DNA damage response, by controlling the stability of REV1, a protein involved in the DNA damage-tolerance mechanisms, responsible for the replication after DNA damage [99]. Accordingly, CDC20-knockdown promoted, in association with acidic culture environment, chromosomal instability in normal lung, colon and epithelial models, resulting in increased survival, metabolic reprogramming and the acquisition of an immortal cancer cell phenotype, characterized by suppression of autophagy and p53-induced apoptosis [98]. In addition, in colorectal cancer, the activation of Wnt/β-catenin signaling during G1 phase, is controlled by CDC20-mediated degradation of conduction [100] and a CDC20-APC/SOX2 axis regulates invasiveness and self-renewal of glioblastoma stem-like cells [101](Fig. 4A-C).

**Fig. 5:**
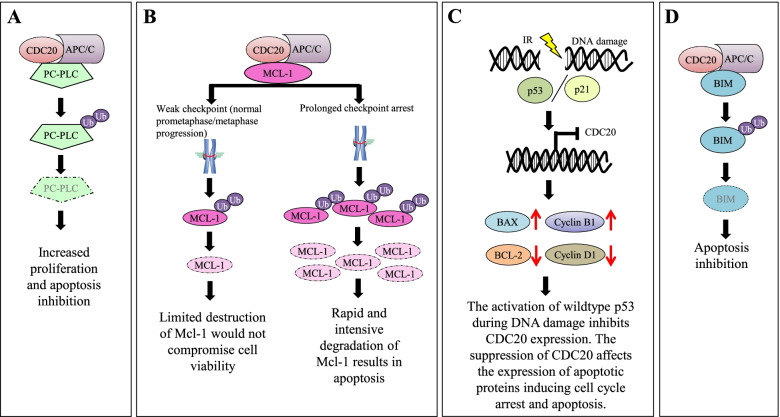
Schematic representation of the role of CDC20 in the regulation of apoptosis. **A** Regulating phosphatidylcholine (PC) cycle. **B** Regulation of MCL-1 activity. **C** Regulation of BCL2 and BAX expression induced by CDC20 following DNA damages. **D** Regulation of BIM activity

CDC20 has been also involved in the apoptotic response, by regulating phosphatidylcholine (PC) cycle [102, 103] and balancing the activity of anti-apoptotic factors, as MCL-1 [104–108] and BCL-2 [108, 109], and pro-apoptotic factors, as BIM [110] (Fig. 5A-D). Regarding PC cycle, CDC20 overexpression modulated the localization of phosphatidylcholine specific phospholipase C (PC-PLC), causing its degradation by the ubiquitin-proteasome pathway [103]. Chen and colleagues also described a role of CDC20-induced degradation of PC-PLC in inducing apoptosis in hepatocellular carcinoma models [102]. Focusing on pro-apoptotic factors, Wan et al. recently reported that BIM is physiologically reduced during mitosis, when APC/C^CDC20^ is active, and that CDC20 depletion allows a significant up-regulation of BIM, activating the DNA damage-induced apoptosis of cancer cells [110]. Regarding MCL-1 dynamics, APC/C^CDC20^ contributes to its degradation during mitosis, and MCL-1 expression levels help distinguish prolonged arrest from normal mitotic events [106, 107]. Sloss and colleagues also described the relationship between MCL-1 and CCNB1 levels, suggesting that MCL-1 competes with CCNB1 for APC/C^CDC20^ binding, thus affecting the rate of CCNB1 degradation and slowering mitotic slippage [105]. Moreover, in colorectal cancer models CDC20 regulated MCL-1 expression levels and its downregulation increased radiosensitivity and induced apoptosis, while BAK, BAX, PUMA, BCL-2, and BCL-xL levels were not affected by the silencing [108]. Conversely, in the HeLa cervix carcinoma model, *CDC20* knockdown resulted in an increased phosphorylation of the anti-apoptotic BCL-2 and BCL-xL proteins and MCL-1 degradation, promoting CDK1 signaling activation and apoptosis [111]. The overexpression of BCL-2 or BCL-xL had a protective role on cell death mediated by *CDC20* downregulation. In breast cancer models CDC20-depleted cells underwent mitotic arrest and were primed to die by apoptosis, which was, at least in part, dependent of BCL-xL phosphorylation on serine 62 residue [112]. Therefore, pharmacological or genetic Bcl-xL (but not BCL-2) silencing, during mitotic arrest was able to induce caspase and Bax-dependent apoptosis.

Recently, CDC20 expression has been also associated with immune infiltration in cancer. A positive correlation has been demonstrated with the infiltration of cancer-associated fibroblasts and myeloid-derived suppressor cells across several cancer types [10], with an immune risk score based on enrichment of 2 T helper cells, memory B cells and plasmacytoid dendritic cells in lower-grade glioma [113], with the infiltration of CD8^+^ T cells, monocytes [114, 115], exhausted T cells [114], CD4^+^ T cells, regulatory T cells, B lymphocytes, and natural killer cells [115] in hepatocellular carcinoma.

In summary, CDC20 is involved in a number of biological and tumor-related functions, that deserve further investigation in the field of hematological malignancies.

### Role of CDC20 in hematological malignancies

**Fig. 6:**
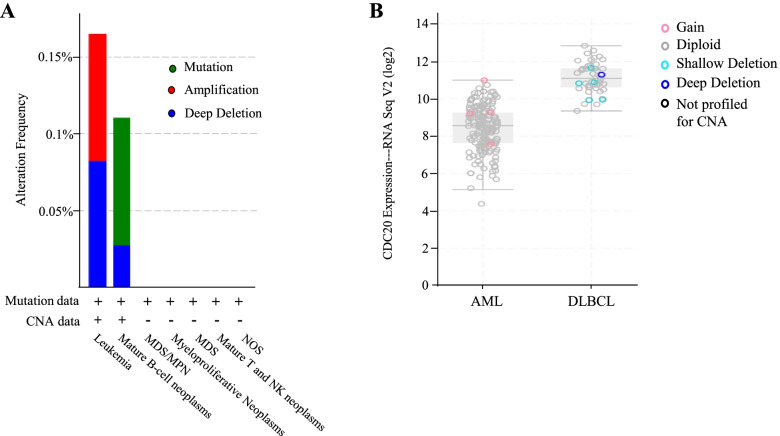
CDC20 alterations across hemato-oncological patients. **A** Frequency and distribution of *CDC20* mutations and copy number alterations (CAN) in hematological malignancies. **B** Effect of CNA on *CDC20* mRNA level in AML and DLBCL cases

Similarly to other SAC genes, *CDC20* is rarely mutated across cancers [9]. Across 6786 onco-hematological cases including pediatric and adult acute lymphoblastic leukemia (ALL) and acute myeloid leukemia (AML), chronic lymphocytic leukemia (CLL), non-Hodgkin lymphomas, myelodysplastic syndrome (MDS) and myeloproliferative neoplasms (MPN), only 3 missense mutations (*G284V, E413G* and *A128T*) have been reported (Fig. 6A, https://www.cbioportal.org). All three mutations reported above have been identified the diffuse large B cell lymphoma (DLBCL) cohort. Moreover, copy number alterations also occur rarely and have been detected in AML and DLBCL, with a frequency lower than 0.2% (https://www.cbioportal.org). They include both amplifications and deletions, with concordant consequences on the gene expression levels in most cases (Fig. 6B). However, copy number losses are quite unexpected based on the pro-tumorigenic role of CDC20, thus suggesting that structural alterations of the gene can be secondary and passenger events with a minor effect on its transcriptional and functional regulation. Despite the low frequency of genetic alterations, CDC20 overexpression and functional deregulation are common events in hematological malignancies, as discussed in the following section (Table 2).

**Table 2:** Deregulated expression of CDC20 and its involvement as prognostic and therapeutic target in hematological malignancies

Cancer type	Evidence in primary samples	Impact on prognosis	Pre-clinical studies	Ref.
ATL	*CDC20* over-expression in ATL samples compared with normal CD4^+^ T cells. Aberrant activation of APC/C^CDC20^ induced by Tax			[116, 117]
AML	CDC20 over-expression in aneuploid and complex karyotype patients.			[118, 119]
CLL	*CDC20* over-expression in aggressive subtypes (U-CLL and CD38^+^ CLL).			[120, 121]
CML	CDC20 stabilization induced by CDH1down-regulation in imatinib-resistance patients			[122]
DLBCL	*CDC20* over-expression	Inferior OS	proTAME induces prolonged metaphase and caspase-dependent apoptosis. Combination of proTAME with Apcin, doxorubicin and venetoclax show synergic effects.	[123, 124]
MCL	*CDC20* over-expression	Inferior OS	proTAME induces prolonged metaphase and caspase-dependent apoptosis. Combination of proTAME with Apcin, doxorubicin and venetoclax show synergic effects.	[124, 125]
MDS	*CDC20* over-expression in high-risk patients	Shorter RFS and inferior OS		[126–128]
MM	CDC20 over-expression in cell lines and high-risk patients	Inferior OS	proTAME treatment induced G2/M arrest and increased apoptosis. Combination with etoposide and doxorubicin, vincristine or melphalan potentiated proTAME effect.	[129–132]

### Lymphoid neoplasms

#### Acute lymphoblastic leukemia

Few data are available on the role of CDC20 in ALL pathogenesis and drug-induced changes of its expression. In the Jurkat T-ALL model, APC/C^CDC20^ was reported to recognize and bind the DEAD-box sequences on the RUNX1 transcription factor, thus inducing its proteosomal degradation, during G2/M-G1 transition [133]. Likewise, APC/C^CDC20^ is responsible for CKS1-mediated degradation of MLL [134], a lysine methyltransferase relevant to hematopoiesis, during the late M phase of the cell cycle, by targeting its N-terminal domain [135]. This assures a correct cell cycle execution, together with SCF^Skp2^ that exerts the same function in the S phase. The expression of the *MLL*-*AF4*, *MLL*-*AF9*, *MLL*-*ENL* and *MLL*-*ELL* fusion transcripts, that characterize ALL and/or AML, confers resistance to MLL degradation mediated by the cell cycle ubiquitin/proteasome system. Moreover, ALL cells exhibit synergic inhibition of proliferation and reduction of viability after combined treatment with arsenic trioxide (ATO) and paclitaxel (PTX) that act through the induction of mitotic arrest and activation of the spindle checkpoint [136]. In particular, ATO/PTX treatment increased the activity of CDK1 resulting in higher phosphorylation of BUBR1 and subsequent formation of the inhibitory checkpoint complex BUB1R/CDC20 that prevented the onset of anaphase.

#### Adult T cell leukemia/lymphoma

Adult T-cell leukemia/lymphoma (ATL) is a CD4^+^ T-cell malignancy caused by infection from human T-cell leukemia virus type 1 (HTLV-1). The progression from infection to malignant transformation has not been fully described, but it has been linked to the activation of the viral trans-activator/oncoprotein, Tax. Tax mediates activation of viral transcription and alters cellular mechanisms in a pleiotropic manner inducing NF-κB activation, cell cycle perturbation and cell transformation [137]. Liu and colleagues found that Tax activation perturbs mitotic entry and G2/M arrest in *S. cerevisiae*, rodent, and human cells leading impaired chromosome segregation and causing severe aneuploidy [138]. The study showed evidence that the mitotic defects caused by Tax are associated with a premature and drastic reduction in Securin and Cyclin B1 levels mediated by APC/C^CDC20^, supporting the idea that Tax promotes aberrant activation of APC/C^CDC20^ to avoid the block of mitotic exit and progression of aneuploid cells, highly represented in ATL [116]. Accordingly, *CDC20* is a hub gene in the protein-protein interaction network of differentially expressed genes between ATL samples and normal CD4^+^ T cells [117].

#### Lymphomas

*CDC20* was also reported as a hub protein among tumor-associated genes in DLBCL [123]. Indeed, in different studies it has been found that high *CDC20* expression correlates with poor overall survival (OS) (*P* = 0.0058 [124]; *P* = 0.0247 [139]) and higher risk of death (hazard ratio, HR = 2.4 [124]) in DLBCL patients. Moreover, a superior sensitivity in prognosis prediction was obtained by combining the expression levels of *CDC20* and *PTGDS2*, another hub gene that is downregulated in DLBCL [123]. Deregulated expression of CCNA, CCNB1 and CDC20 conferred to B-cell lymphoma cells the ability to aberrantly bypass the mitotic arrest, as demonstrated in the IgHμ-*HOX11* transgenic mouse [140]. CDC20 expression was regulated by the MDM2-p53 pathway in DLBCL [139]. Indeed, MDM2 silencing restored p53 expression and reduced CDC20 protein level in DLBCL cell lines.

Recent studies showed that *CDC20* is highly expressed not only in DLBCL but also in mantle cell lymphoma (MCL) [124, 125]. Functional enrichment analysis performed on gene expression data revealed that *CDC20* is among the top five altered genes involved in the development of MCL and it is significantly associated with shorter OS (5-year OS around 10 and 60% in CDC20 high and low, respectively; *P* = 2.623e^− 11^ [125]).

#### Chronic lymphocytic leukemia

In CLL, high *CDC20* expression has been reported in the high-risk category characterized by unmutated *IGHV* (U-CLL) [120]. Indeed, compared with *IGHV* mutated (M-CLL) cases, the more aggressive U-CLL subtype exhibited an increased expression of cell cycle genes, including *ATF2, CCNB2, CDC20, CDC25A, CREB1, E2F4, ESR1, FOXM1, MKI67, MYC, POU2F2, RBL2, SP3, TYMS, UBE2C, VRK1*. CDC20 was also associated with CD38 expression, another marker of disease aggressiveness [121]. Moreover, primary CD38^+^ B-CLL samples had an increased level of APC/C subunit 5 (APC/C 5), which controls some regulatory sub-functions of the APC/C complex [121]. As demonstrated in Drosophila models, APC/C 5 mediated the “wait” signal from the SAC and, in presence of mutations disrupting that signal, mitotic cells prematurely advanced through chromatid segregation and anaphase [141]. This evidence suggests that the overexpression of APC/C 5 observed in CD38^+^ CLL cells could represent an alternative strategy adopted to mimic the effects of CDC20 overexpression.

#### Multiple myeloma

The genome of multiple myeloma (MM) patients is highly unstable and is characterized by chromosome translocations and aneuploidy, affecting the disease outcome [142]. The high degree of aneuploidies suggested that MM cells exhibit a weakened SAC activity that allows them to tolerate gains or losses of a small number of chromosomes. Indeed, MM cell lines generally expressed lower levels of some SAC components (*AURKC*, *PLK2*, *PLK3*) compared to normal plasma cells and higher levels of others, including *CDC20*, and were able to bypass the SAC-mediated arrest when challenged with nocodazole [129]. High levels of *CDC20* transcript were also confirmed in primary cells from high-risk MM patients [130], that also displayed elevated expression of *BUB1B* [131] together with reduced levels of *CDH1* [143], that sustain MM cell proliferation. Indeed, *CDC20* knockdown reduced the viability of MM cell lines, by inducing cell growth arrest and accumulation of the APC/C^CDC20^ substrate CCNB1 [132]. Moreover, high *CDC20* expression was associated with inferior OS both per se (*P* = 1.08e-05 and *P* = 0.00619 in TT2-cohort and HM-cohort, respectively [130]) and in combination with *BUB1B* and *CCNB* levels (*P* < 0.05 [131]).

### Myeloid neoplasms

#### Myelodysplastic syndrome

Emerging evidence shows that MDS patients harbor deregulated expression of several components of SAC machinery. In particular, MDS patients with hypercellular and normocellular bone marrow, reflecting a more aggressive disease, had higher expression of SAC components in comparison with those characterized by hypocellular bone marrow [126]. Moreover, high levels of *CDC20* and *MAD2* characterized MDS patients with severe thrombocytopenia and complex karyotypes [127]. Notably, the higher expression of both genes was associated with a significantly poorer OS in MDS patients (*P* = 0.013) [127].

#### Acute myeloid leukemia

More than 20% of AML patients have defects in chromosome segregation, also supported by an altered activity of the SAC components, caused by BUB1 deregulation, BUBR1 repression, or aberrant expression of MAD2 and CDC20 [9, 144, 145], that was further exacerbated by decitabine treatment in AML cell lines [144]. We have recently shown that CDC20 is upregulated both at transcript and protein level in aneuploid compared with euploid AML and a 3-gene signature including high *CDC20* and *PLK1* and low *RAD50* expression was able to discriminate the aneuploid from euploid cases [118]. In addition, complex karyotype AML, which includes a number of aneuploid cases, was enriched for a G2/M checkpoint gene signature, including *CDC20* [119]. CDC20 was reported to interact with proteins playing a crucial role in AML pathogenesis, including RUNX1 [146], MEIS1, p21 [147] and NUP98 [148]. CDC20 (and CDH1) can target RUNX1 to degradation by APC/C. Binding of CDC20 (but not CDH1) to RUNX1 was mediated by phosphorylation of the target at serine 276 and 303 residues [146]. Moreover, it has been demonstrated that CDC20-mediated ubiquitination of MEIS1 and p21 participates in the regulation of quiescence in hematopoietic stem cells and leukemia initiating cells [147]. In particular, MEIS1 and p21 degradation was hampered by PPM1K thorough induction of branched chain amino acid catabolism, which in turn resulted in reduced protein ubiquitination by CDC20 and enforced glycolysis and quiescence of AML cells. APC/C^CDC20^ also showed an aberrant interaction with NUP98 fusion oncoproteins [148], a rare pathogenic mechanism in AML that is, however, overrepresented in high-risk pediatric patients [149]. Wildtype NUP98 is a conditional target of APC/C^CDC20^ and the physical interaction is dependent on the phosphorylation of a PEST sequence within NUP98 C-terminal domain, which occurs prior to mitotic entry [150]. The peptidyl-prolyl isomerase PIN1 then induces NUP98 conformational changes driving its dissociation from APC/C^CDC20^ during mitosis. Conversely, Salsi et al. demonstrated that NUP98 fusion oncoproteins bind APC/C^CDC20^ during mitosis, through the NUP98 GLEBS-like domain in the absence of the RAE1 partner protein. This interaction led to BUBR1 displacement and consequent attenuation of the SAC [148], that could be restored by CDC20 or MAD2 overexpression [150].

#### Chronic myeloid leukemia

Tyrosine kinase inhibitors, which have changed the management of chronic myeloid leukemia (CML) patients during the last 10 years [151], control cell cycle and apoptosis through several mechanisms, including the regulation of CDH1 levels [122]. It has been shown that CDH1 expression is significantly lower in imatinib-resistant CML blast crisis patients compared with imatinib-sensitive ones and its downregulation induced stabilization of SKP2 and CDC20, resulting in increased proliferation and genomic instability, with the formation of multinucleated cells, suggesting a role of CDC20 in therapy resistance [122].

### CDC20 targeting with specific inhibitors in hematological malignancies

Due to the potential oncogenic role of CDC20, different chemical compounds and inhibitors have been developed and tested for their efficacy as antineoplastic agents: tosyl-L-arginine methyl ester (TAME) and its pro-drug (pro-TAME), APC inhibitor (Apcin), Withaferin A, N-alkylated amino acid-derived (NAHA), Ganodermanontriol, Genistein, CARP-1 functional mimetic 4 (CFM-4) and 6-brominated coumarin hydrazide-hydrazone derivative (BCHHD), that have been extensively revised by Wang et al [30]. Among them, Apcin and pro-TAME have been identified as selective CDC20 and APC/C^CDC20^/APC/C^CDH1^ inhibitors [152], respectively, and are currently under preclinical investigation for their efficacy against different cancer subtypes, including hematological malignancies.

#### Apcin

**Fig. 7:**
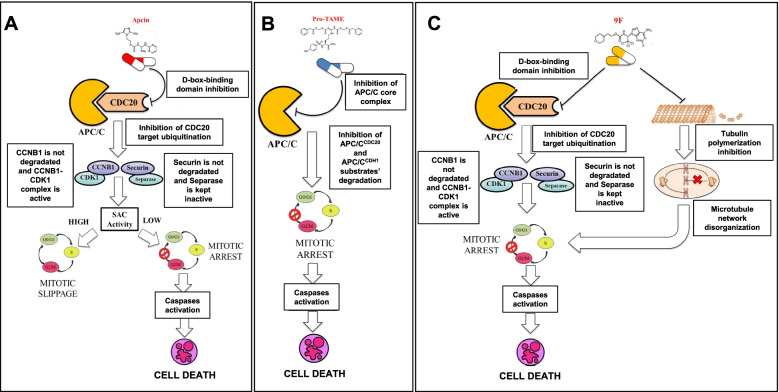
Schematic representation of CDC20 inhibition strategies tested in hematological malignancies. **A** The small molecule Apcin prevents the substrate recognition capacity of APC/C^CDC20^ leading to the stabilization of CDC20 substrates that could result in mitotic arrest or mitotic slippage based on intracellular SAC activity. **B** Pro-Tame binds in a competitive manner the APC/C core complex preventing its association with CDC20 that results in mitotic arrest through stabilization of CCNB1. **C** The compound 9f, like Apcin, inhibits CDC20 downstream activity leading to mitotic arrest. It has also been shown that 9f inhibits tubulin polymerization and compromises microtubule network organization, causing cell cycle arrest and inducing apoptosis

Apcin is a small molecule that binds in a competitive manner the D-box-binding domain of CDC20 thus preventing its substrate recognition capacity and inhibiting the ubiquitination of CDC20 targets [152] (Fig. 7A). Surprisingly, it has been recently observed that Apcin had a paradoxical effect on tumor cell lines: it induces mitotic arrest (which is the predicted effect of an APC/C^CDC20^ inhibitor) or mitotic slippage depending on the low or high SAC activity, respectively [153]. Regarding the mitotic slippage, Apicin interacts with the D-box-binding domain on CDC20 which is essential for both substrate ubiquitination and mitotic checkpoint complex-dependent APC/C inhibition through BUBR1 interaction. In the field of hematological malignancies, the efficacy of Apcin has been tested in DLBCL and MM models. In DLBCL, Apcin significantly reduced cell viability and proliferation and induced cell cycle arrest in G2/M phase and apoptosis of OCI-Ly3 and OCI-Ly10 lines [139]. In vivo models confirmed that Apcin treatment dampened CDC20 expression and inhibited the tumor growth in NOD/SCID mice bearing OCI-Ly10 xenografts [139]. Apcin also showed an activity in MM models, with minor effects as a single agent, but a higher efficacy in combination with pro-TAME, in terms of apoptosis induction [130].

#### Pro-TAME

pro-TAME is a small molecule that mimics the IR motif of CDC20 and CDH1 involved in their recruitment to the APC/C. It binds the APC/C core complex in a competitive manner and prevents its association with APC/C activators [154] (Fig. 7B), leading to the inhibition of APC/C^CDC20^ and APC/C^CDH1^ substrates’ degradation and mitotic arrest [155]. Pro-TAME has also been tested in combination with Apcin, showing a synergistic effect on the stabilization of CCNB1, securin, CCNA2 and NEK2A together with a significant increase of the mitotic fraction [152].

In MCL and DLBCL cellular models proTAME induced metaphase arrest, resulting in accumulation of the APC/C^CDC20^ substrate CCNB1, together with phosphorylation-mediated inactivation of the anti-apoptotic factors BCL-2 and BCL-xL, reduction of cell viability and activation of caspase-3 dependent apoptosis [124]. Pro-TAME efficacy was confirmed in primary cells from MCL and DBCL patients. In addition, proTAME strongly synergized with Apcin and clinically relevant drugs, including doxorubicin and venetoclax in lymphoma cellular models.

Consistently with the high CDC20 expression in MM, treatment of cell lines and primary cells with proTAME resulted in the stabilization of CCNB1 and cell cycle arrest at G2/M phase [130, 132]. Moreover, cells treated with proTAME showed cleavage of caspase 3, 8, 9 and PARP, and accumulation of the pro-apoptotic protein BIM, leading to apoptosis [130, 132]. Cell death induction in both MM cell lines and primary samples was further exacerbated by the combinations of proTAME with topoisomerase inhibitors, etoposide and doxorubicin, especially when proTAME treatment was preceding the administration of the other drugs and in association with the microtubule inhibitor vincristine or the chemotherapy agent melphalan [130].

## Conclusion

In line with evidence from solid cancer, CDC20 overexpression also plays a critical oncogenic role in hematological malignancies. The available data gathered in this review revealed that the up-regulation of CDC20 is associated with inferior OS in different hematological malignances. In agreement with its potential role as prognostic marker, higher expression of CDC20 was observed in high-risk MM, CLL, MDS and AML patients. Moreover, over-expression of CDC20 has been reported as a biomarker of resistance to TKI therapy in CML patients. In line with these observations, studies performed in DLBCL, MCL and MM cells treated with Apcin or proTAME, or their combination, demonstrated that targeting CDC20 is a promising therapeutic strategy in hemato-oncology. Indeed, it has been shown that CDC20 inhibitors significantly potentiate the efficacy of conventional therapeutic agents in different hematological malignances. In addition, novel therapeutic combinations based on the synthetic lethality mechanisms could be explored. For example, Apcin effectiveness is enhanced in cells carrying defective sister chromatid cohesion, that also characterize a subgroup of AML patients [156], as shown by using cellular models of Warsaw breakage syndrome with defective function of the DNA helicase DDX11 [157]. In addition, the correlation between CDC20 expression and infiltration of immune cells, including those inducing tolerance, led us to hypothesize that targeting CDC20 may reinforce the immune response and also synergize with immunomodulatory drugs in patients expressing high CDC20 levels.

Further development towards clinical application of CDC20 inhibitors is hampered by the poor bioavailability of the compounds, because of the high dosage needed to achieve a therapeutic response. This evidence and the current knowledge provided the rationale for the development of other specific inhibitors. We have synthesized and tested novel tryptamine derivatives bearing aminopyrimidyl- or imidazolyl- moieties, which are also present in Apcin [158]. In particular, compound 9, characterized by 2-aminopyrimidyl- and trichloroethyl- moieties, similarly to those in Apcin, showed a preferential efficacy in hematology compared with solid tumor models, and significantly reduced the growth of AML and ALL cells. Moreover, Huang and colleagues synthesized a series of 2,2,2-trichloro-1-aryl carbamate derivatives starting from the modification of Apcin structure [159]. They identified two compounds, namely 7d and 9f showing a higher efficacy compared with Apcin in terms of mitotic arrest and apoptosis induction, which occurred through stabilization of CCNB1 and activation of caspase-3 and PARP, respectively. Interestingly, the most potent one, compound 9f, also played additional functions, as it inhibited cell migration, invasion and tubulin polymerization and it disorganized the microtubule network. Thus, the increased compound efficacy may be related to the dual activity, in line with a recent study reporting that inhibition of APC/C^CDC20^ enhances the sensitivity of cancer cells to microtubule interfering agents [160] (Fig. 7C).

Overall, this evidence proves a growing therapeutic interest in CDC20 targeting in hematological malignancies, which will promote novel studies towards the development of better combination strategies, the identification of patients’ cohorts that will mostly benefit of them and the definition of optimal therapeutic windows.

## Supplementary Information

**Additional file 1.**

## Data Availability

Data sharing not applicable to this article as no datasets were generated or analysed during the current study.

## Abbreviations

ABL1: Abelson
AML: Acute myeloid leukemia
Apc1-loop^500^: Apc1 loop domain
APC/C: Anaphase promoting complex/cyclosome
ATF2: Activating Transcription Factor 2
ATL: Adult T-cell leukemia/lymphoma
Bcl-2: B-cell lymphoma 2
Bcl-xL: B-cell lymphoma-extra large
BCR: Breakpoint cluster region
BIM: Bcl-2-like protein 11
BM: Bone marrow
BUB1: BUB1 Mitotic Checkpoint Serine/Threonine Kinase
BUB3: BUB3: Mitotic Checkpoint Protein
BUBR1/BUB1B: BUB1 Mitotic Checkpoint Serine/Threonine Kinase B
CCNB2: Cyclin B2
CDC20: Cell division cycle 20 homologue
CDC25A: Cell division cycle 25 A
CDH1: CDC20 homologue 1
CDK1: Cyclin-dependent kinase 1
CEMP-F: Centromere protein F
CLL: Chronic lymphocytic leukemia
CML: Chronic myeloid leukemia
CREB1: CAMP Responsive Element Binding Protein 1
DLBCL: Diffuse large B-cell lymphoma
DNA: Deoxyribonucleic acid
E2F4: E2F Transcription Factor 4
EMI 1: Early mitotic inhibitor1
ESR1: Estrogen Receptor 1
FOXM1: Forkhead Box M1
HTLV-1: Human T-cell leukemia virus type 1
HSC: Hematopoietic stem cells
IGHV: Immunoglobulin heavy chain variable region
IPSS-R: International Prognostic Scoring System
JUN: AP-1 Transcription Factor Subunit
KMN: Knl1 complex, the Mis12 complex and the Ndc80 complex
KNL1: Kinetochore Scaffold 1
TKIs: Tyrosine kinase inhibitors
MAD1/MAD1L1: MAD1 Mitotic Arrest Deficient Like 1
MAD2/MAD2L1: Mitotic Arrest Deficient 2 Like 1
MCC: Mitotic checkpoint complex
MCL: Mantle cell lymphoma
MCL-1: Myeloid cell leukemia 1
M-CLL: Mutated-Chronic lymphocytic leukemia
MDS: Myelodisplastic syndromes
MIM: MAD2-interacting motif
MKI67: Marker Of Proliferation Ki-67
miR: micro RNA
mRNA: messenger RNA
MIS12: Kinetochore Complex Component
MM: Multiple myeloma
MPS1: TTK Protein Kinase
MYC: Myelocytomatosis oncogene
NEK2A: Never in Mitosis (NIMA) Related Kinase 2A
NDC80: NDC80, Kinetochore Complex Component
OS: Overall survival
PARP: Poli ADP-ribosio polimerasi
PB: Peripheral blood
PC-PLC: Phosphatidylcholine specific phospholipase C
PLK1: Polo Like Kinase 1
POU2F2: POU Class 2 Homeobox 2
p21: Cyclin-dependent kinase inhibitor 1
RAD50: Double Strand Break Repair Protein
RAF: Proto-Oncogene, Serine/Threonine Kinase
RAS: Rat Sarcoma-oncogene
RASSF1A: Ras association domain family 1 isoform A
RBL2: RB Transcriptional Corepressor Like 2
RFS: Recurrence-free survival
SAC: Spindle assembly checkpoint
SP3: Specificity Protein 3
STAT: Signal Transducer And Activator Of Transcription
Tax: Trans-activator/oncoprotein
TBK1: Tank Binding Kinase 1
TYMS: Thymidylate Synthetase
TP53/p53: Tumor protein 53
TRP: Tetratricopeptide repeat
UBE2C: Ubiquitin Conjugating Enzyme E2 C
U-CLL: Unmutated-Chronic lymphocytic leukemia
USP44: Ubiquitin-specific protease 44
VRK1: Vaccinia-related kinase 1
