# Supplementary material for: CDC20 in and out of mitosis: a prognostic factor and therapeutic target in hematological malignancies
Source: J Exp Clin Cancer Res. 2022 Apr 30;41:159. doi: 10.1186/s13046-022-02363-9 (PMC9055704; doi:10.1186/s13046-022-02363-9)
Supplement: Supplementary file 1 — Additional file 1. [file 13046_2022_2363_MOESM1_ESM.docx]

**SUPPLEMENTARY TABLE**

**Table S1. Pathway enrichment analysis of CDC20 interacting proteins.**

| **Macro-pathway** | **DB** | **Term** | **Adj-p** | **Genes** |
| --- | --- | --- | --- | --- |
| Cell cycle | KEGG 2021 | Cell cycle | 3.92E-54 | ANAPC13;CDKN1A;HDAC2;PCNA;HDAC1;CUL1;BUB1B;TTK;ANAPC10;ANAPC11;CDC20;CCNB1;FZR1;CDC23;PTTG1;CDC26;MYC;CDC27;E2F1;EP300;BUB3;SKP2;BUB1;CREBBP;TGFB1;ANAPC7;PLK1;CDC6;CDC25A;CDC25B;CCNA2;MAD2L2;CCNA1;TFDP1;CDC16;CDK2;CDK1;ANAPC4;ANAPC5;ANAPC1;MAD1L1;ANAPC2;MAD2L1 |
|  | GO 2021 | regulation of mitotic cell cycle phase transition | 2.05E-47 | ANAPC15;ANAPC16;CUL3;CUL1;BUB1B;ANAPC10;ANAPC11;AURKA;CDC20;CCNB1;FZR1;CDC23;TUBA1A;UBB;PPP2R1A;CDC26;CDC27;UBC;E2F1;NEK2;FBXO5;BUB3;BTRC;NINL;ANAPC7;UBE2C;TUBB;PLK1;MAD2L1BP;CDC6;TUBG1;TUBB4B;TUBA4A;SIRT2;HAUS1;UBE2S;HECW2;CDC16;CDK2;CDK1;ANAPC4;ANAPC5;ANAPC1;ANAPC2;MAD2L1 |
|  | GO 2021 | anaphase-promoting complex-dependent catabolic process | 1.92E-42 | ANAPC15;ANAPC16;CUL3;BUB1B;ANAPC10;AURKB;ANAPC11;AURKA;CDC20;CCNB1;FZR1;CDC23;PTTG1;UBB;CDC26;CDC27;UBC;FBXO5;BUB3;SKP2;ANAPC7;UBE2C;PLK1;FBXO31;UBE2S;CDC16;CDK2;CDK1;ANAPC4;ANAPC5;ANAPC1;ANAPC2;MAD2L1 |
|  | GO 2021 | regulation of exit from mitosis | 1.02E-36 | ANAPC15;ANAPC16;ANAPC7;UBE2C;BUB1B;MAD2L1BP;ANAPC10;SIRT2;ANAPC11;CDC20;CDC23;UBB;UBE2S;CDC26;CDC27;UBC;CDC16;ANAPC4;NEK2;ANAPC5;BUB3;ANAPC1;ANAPC2;MAD2L1 |
|  | GO 2021 | regulation of mitotic cell cycle | 4.83E-35 | APP;ANAPC15;ANAPC16;CUL1;BUB1B;FBXO43;ANAPC10;ANAPC11;CKS1B;CDC20;CCNB1;FZR1;CDC23;CDC26;CDC27;HSF1;NEK2;FBXO5;BUB3;BTRC;TGFB1;ANAPC7;UBE2C;USP22;PLK1;CDC25B;UBE2S;CDC16;CDK1;CKS2;ANAPC4;ANAPC5;ANAPC1;ANAPC2;TP73;MAD2L1 |
|  | GO 2021 | mitotic cell cycle phase transition | 4.85E-31 | CDKN1A;USP37;CUL3;CCNF;CUL1;PHF8;AURKA;CCNB1;CDC23;TUBA1A;PPP2R1A;MYC;CDC27;E2F1;NEK2;SKP2;BTRC;NINL;ANAPC7;UBE2C;TUBB;PLK1;TUBG1;TUBB4B;TUBA4A;CDC25A;CDC25B;HAUS1;CCNA2;CCNA1;UBE2S;CDC16;CDK2;CDK1;ANAPC1 |
|  | GO 2021 | regulation of cell cycle phase transition | 2.54E-30 | CDKN1A;ANAPC15;ANAPC16;CUL1;BUB1B;ANAPC10;ANAPC11;CDC20;CCNB1;FZR1;CDC23;CDC26;CDC27;FBXO5;BUB3;BTRC;ANAPC7;UBE2C;PLK1;UBE2S;CDC16;CDK1;ANAPC4;ANAPC5;ANAPC1;ANAPC2;MAD2L1 |
|  | GO 2021 | regulation of mitotic metaphase/anaphase transition | 3.22E-22 | ANAPC15;ANAPC7;UBE2C;CUL3;PLK1;CDC6;ANAPC11;CCNB1;CDC23;HECW2;CDC27;CDC16;ANAPC4;ANAPC5;FBXO5 |
|  | GO 2021 | G2/M transition of mitotic cell cycle | 6.14E-18 | CDKN1A;TUBB;PLK1;CUL1;TUBG1;TUBB4B;CDC25A;TUBA4A;CDC25B;HAUS1;AURKA;CCNA2;CCNB1;TUBA1A;PPP2R1A;CDK2;CDK1;NEK2;BTRC;SKP2;NINL |
|  | GO 2021 | cell cycle G2/M phase transition | 6.73E-18 | CDKN1A;TUBB;PLK1;CUL1;TUBG1;TUBB4B;CDC25A;TUBA4A;CDC25B;HAUS1;AURKA;CCNA2;CCNB1;TUBA1A;PPP2R1A;CDK2;CDK1;NEK2;BTRC;SKP2;NINL |
|  | GO 2021 | negative regulation of mitotic metaphase/anaphase transition | 2.78E-17 | CDC20;MAD2L2;PLK1;BUB1B;TTK;BUB3;FBXO5;TRIP13;BUB1;ZWINT;MAD1L1;MAD2L1 |
|  | GO 2021 | positive regulation of mitotic cell cycle phase transition | 6.53E-16 | ANAPC7;UBE2C;CUL3;CDC25A;CDC25B;ANAPC11;CCNB1;CDC23;TFDP1;CDC27;CDC16;CDK1;ANAPC4;ANAPC5;FBXO5 |
|  | GO 2021 | mitotic spindle assembly checkpoint signaling | 1.32E-15 | CDC20;MAD2L2;PLK1;BUB1B;TTK;BUB3;TRIP13;BUB1;ZWINT;MAD1L1;MAD2L1 |
|  | GO 2021 | mitotic spindle checkpoint signaling | 1.32E-15 | CDC20;MAD2L2;PLK1;BUB1B;TTK;BUB3;TRIP13;BUB1;ZWINT;MAD1L1;MAD2L1 |
|  | GO 2021 | spindle assembly checkpoint signaling | 1.32E-15 | CDC20;MAD2L2;PLK1;BUB1B;TTK;BUB3;TRIP13;BUB1;ZWINT;MAD1L1;MAD2L1 |
|  | GO 2021 | regulation of mitotic sister chromatid separation | 3.24E-15 | CDC23;ANAPC7;PTTG1;UBE2C;HECW2;PLK1;CDC16;CDC27;CDC6;ANAPC1 |
|  | GO 2021 | G1/S transition of mitotic cell cycle | 2.19E-13 | CDT1;CDKN1A;USP37;PCNA;CUL3;CUL1;CDC6;CDC25A;PHF8;CCNA1;TFDP1;MYC;CDK2;E2F1;FBXO5 |
|  | GO 2021 | regulation of G2/M transition of mitotic cell cycle | 3.39E-13 | TUBB;PLK1;CUL1;TUBG1;TUBB4B;CDC25A;TUBA4A;CDC25B;HAUS1;AURKA;CCNB1;TUBA1A;PPP2R1A;CDK2;CDK1;NEK2;FBXO5;NINL |
|  | GO 2021 | positive regulation of metaphase/anaphase transition of cell cycle | 1.56E-12 | CDC23;ANAPC7;CUL3;CDC16;CDC27;ANAPC4;ANAPC5;ANAPC11 |
|  | GO 2021 | positive regulation of mitotic metaphase/anaphase transition | 1.56E-12 | CDC23;ANAPC7;CUL3;CDC16;CDC27;ANAPC4;ANAPC5;ANAPC11 |
|  | GO 2021 | positive regulation of mitotic sister chromatid separation | 1.56E-12 | CDC23;ANAPC7;CUL3;CDC16;CDC27;ANAPC4;ANAPC5;ANAPC11 |
|  | GO 2021 | microtubule cytoskeleton organization involved in mitosis | 4.96E-12 | PLK1;BUB1B;TTK;TUBG1;ZWINT;AURKB;AURKA;CDC20;CCNB1;KIF18A;XPO1;NUP98;BUB3;BUB1;MAD1L1;MAD2L1 |
|  | GO 2021 | positive regulation of DNA biosynthetic process | 2.19E-11 | CCT3;CCT6A;CCT2;PCNA;MYC;TCP1;NEK2;CCT8;CCT7;AURKB;CCT5;CCT4 |
|  | GO 2021 | regulation of cell cycle G2/M phase transition | 6.39E-11 | TUBB;PLK1;TUBG1;TUBB4B;TUBA4A;HAUS1;AURKA;TUBA1A;PPP2R1A;CDK2;CDK1;NEK2;NINL |
|  | GO 2021 | mitotic spindle organization | 9.68E-11 | PLK1;BUB1B;TTK;TUBG1;ZWINT;AURKB;AURKA;CDC20;CCNB1;KIF18A;XPO1;NUP98;BUB3;BUB1;MAD1L1;MAD2L1 |
|  | GO 2021 | positive regulation of mitotic nuclear division | 1.59E-09 | CDC23;ANAPC7;CUL3;CDC27;CDC16;ANAPC4;ANAPC5;ANAPC11;AURKA |
|  | GO 2021 | cell cycle G1/S phase transition | 1.49E-08 | CCNA2;CDKN1A;USP37;MYC;CUL3;CDK2;CUL1;E2F1;CDC25A;PHF8 |
|  | GO 2021 | metaphase/anaphase transition of cell cycle | 1.52E-08 | CDC23;ANAPC7;CDC16;CDC27;ANAPC1 |
|  | GO 2021 | metaphase/anaphase transition of mitotic cell cycle | 1.52E-08 | CDC23;ANAPC7;CDC16;CDC27;ANAPC1 |
|  | GO 2021 | regulation of DNA replication | 5.11E-08 | CCNA2;PPP2CA;CDT1;USP37;PCNA;PPP2R1A;GMNN;FBXO5;CDC6 |
|  | GO 2021 | regulation of metaphase/anaphase transition of cell cycle | 1.14E-07 | CDC23;UBE2C;HECW2;PLK1;CDC6 |
|  | GO 2021 | regulation of cell cycle | 1.14E-07 | CDKN1A;TGFB1;RPL23;GMNN;PLK1;CDC25A;SIRT2;CKS1B;RASSF1;CCNB1;CDC26;MYC;CKS2;EP300;FBXO5;SKP2;TP73 |
|  | GO 2021 | regulation of transcription involved in G1/S transition of mitotic cell cycle | 4.83E-07 | CDT1;CCNA1;PCNA;TFDP1;E2F1;FBXO5;CDC6 |
|  | GO 2021 | regulation of cyclin-dependent protein serine/threonine kinase activity | 2.10E-06 | CCNA2;CCNA1;CDKN1A;HHEX;CCNB1;CCNF;PLK1;CDC6;CDC25A |
|  | GO 2021 | positive regulation of mitotic cell cycle | 3.96E-06 | APP;CCNB1;CUL3;USP22;HSF1;CDC25B |
|  | GO 2021 | mitotic G2/M transition checkpoint | 4.85E-06 | CDKN1A;FZR1;PLK1;CDK1;CLSPN;CDC6;CHFR |
|  | GO 2021 | mitotic nuclear membrane organization | 1.08E-05 | PPP2CA;CCNB1;PPP2R1A;IST1;CDK1;NUP98;SIRT2 |
|  | GO 2021 | mitotic nuclear membrane reassembly | 1.08E-05 | PPP2CA;CCNB1;PPP2R1A;IST1;CDK1;NUP98;SIRT2 |
|  | GO 2021 | positive regulation of cell cycle process | 1.09E-05 | CCNB1;CUL3;TAS2R13;CDC6;SIRT2;AURKB;CDC25B;AURKA;MAD2L1 |
|  | GO 2021 | mitotic sister chromatid segregation | 1.17E-05 | CCNB1;KIF18A;CDC23;CUL3;PLK1;CDK1;TUBG1;ZWINT;PHF8 |
|  | GO 2021 | regulation of cyclin-dependent protein kinase activity | 1.52E-05 | CCNA2;CCNA1;CDKN1A;CCNB1;CCNF;CDC6;CDC25A |
|  | GO 2021 | nuclear membrane reassembly | 1.52E-05 | PPP2CA;CCNB1;PPP2R1A;IST1;CDK1;NUP98;SIRT2 |
|  | GO 2021 | chromosome organization | 1.56E-05 | CDT1;HHEX;HDAC2;COPS5;UBB;HDAC1;MYC;UBC;AXIN2 |
|  | GO 2021 | positive regulation of G2/M transition of mitotic cell cycle | 1.92E-05 | CCNB1;CDK1;FBXO5;CDC25A;CDC25B |
|  | GO 2021 | mitotic cell cycle checkpoint signaling | 3.90E-05 | BUB1B;BUB1;AURKB;CHFR;ZWINT |
|  | GO 2021 | positive regulation of cell cycle G2/M phase transition | 3.90E-05 | CCNB1;CDK1;FBXO5;CDC25A;CDC25B |
|  | GO 2021 | cell cycle checkpoint signaling | 6.34E-05 | BUB1B;BUB1;CHFR;ZWINT |
|  | GO 2021 | protein localization to condensed chromosome | 6.34E-05 | CDK1;TTK;BUB3;AURKB |
|  | GO 2021 | positive regulation of cell division | 6.34E-05 | CUL3;TAS2R13;CDC6;AURKB;SIRT2;CDC25B |
|  | GO 2021 | negative regulation of mitotic cell cycle | 9.98E-05 | TGFB1;BUB1B;BUB1;CHFR;ZWINT;MAD2L1 |
|  | GO 2021 | protein localization to kinetochore | 1.19E-04 | CDK1;TTK;BUB3;AURKB |
|  | GO 2021 | regulation of cell cycle process | 1.29E-04 | PLK1;NEK2;FBXO5;FBXO43;BUB1;MAD1L1;AURKB;AURKA |
|  | GO 2021 | mitotic metaphase plate congression | 1.35E-04 | CDT1;CCNB1;KIF18A;CDC23;CUL3;MAD1L1 |
|  | GO 2021 | regulation of cytokinesis | 2.19E-04 | CUL3;PLK1;TAS2R13;CDC6;AURKB;CDC25B;AURKA |
|  | GO 2021 | regulation of mitotic nuclear division | 2.26E-04 | CCNB1;ANAPC15;NEK2;FBXO5;FBXO43;AURKA |
|  | GO 2021 | protein localization to chromosome, centromeric region | 2.28E-04 | CDK1;TTK;BUB3;AURKB |
|  | GO 2021 | positive regulation of cytokinesis | 3.30E-04 | CUL3;TAS2R13;CDC6;AURKB;CDC25B |
|  | GO 2021 | negative regulation of cell cycle process | 4.10E-04 | TFDP1;E2F1;NEK2;FBXO5;FBXO43;AURKB |
|  | GO 2021 | positive regulation of chromosome segregation | 5.29E-04 | CCNB1;CDC6;SIRT2 |
|  | GO 2021 | mitotic nuclear division | 8.20E-04 | KIF18A;UBE2C;UBE2S;PLK1;TUBG1;ZWINT |
|  | GO 2021 | regulation of cell division | 9.28E-04 | MYC;PLK1;TP63;SIRT2;AURKB;AURKA |
|  | GO 2021 | regulation of attachment of spindle microtubules to kinetochore | 9.82E-04 | CCNB1;NEK2;SIRT2 |
| Cell death | GO 2021 | negative regulation of programmed cell death | 7.80E-07 | HSPA9;HDAC2;HSPA5;HDAC1;PLK1;HSPD1;COPS5;HAX1;UBB;DDRGK1;MYC;UBC;PIM1;CDK1;DCPS;MCL1;MAD2L1;HSPA1A |
|  | GO 2021 | regulation of apoptotic process | 1.21E-06 | HDAC2;HDAC1;HSPD1;UBB;DDRGK1;MYC;UBC;PIM1;RPS3;E2F1;SKP2;TP63;MCL1;HSPA9;DAXX;CREBBP;EGLN3;HSPA5;PLK1;COPS5;HAX1;CDK1;TP73;MAD2L1;HSPA1A |
|  | GO 2021 | negative regulation of apoptotic process | 1.94E-05 | HSPA9;YAP1;HDAC2;HSPA5;HDAC1;PLK1;HSPD1;COPS5;HAX1;UBB;DDRGK1;MYC;UBC;PIM1;CDK1;MCL1;MAD2L1;HSPA1A |
| Cell proliferation | GO 2021 | regulation of cell population proliferation | 2.07E-06 | YAP1;APP;CDKN1A;HDAC2;HDAC1;CUL3;TTK;HHEX;CAPNS1;DDRGK1;MYC;HSF1;FBXO5;PBRM1;EGLN3;TGFB1;RPL23;CDC6;AXIN2;SIRT2;CDC25B;CDK2;DNAJA2;FGFR1;HSPA1A |
|  | GO 2021 | positive regulation of cell population proliferation | 3.73E-06 | YAP1;CDKN1A;HDAC2;TGFB1;HDAC1;RPL23;CUL3;TTK;CDC25B;CAPNS1;DDRGK1;MYC;CDK2;HSF1;DNAJA2;PIM1;E2F1;FBXO5;FGFR1 |
|  | GO 2021 | negative regulation of cellular macromolecule biosynthetic process | 9.31E-05 | HSPA8;DAXX;CDKN1A;HDAC2;TGFB1;HDAC1;GMNN;CDC6;YBX1;SIRT2;HDAC6;HHEX;ID1;RPS3;E2F1;BTRC;TP63;TRIM33 |
|  | GO 2021 | regulation of cellular macromolecule biosynthetic process | 5.40E-04 | APP;DAXX;CREBBP;USP37;TRRAP;GMNN;HOXD13;YBX1;CCNA2;PPP2CA;PPP2R1A;E2F1;EP300;FBXO5;EIF2A |
| DNA damage | GO 2021 | mitotic G1 DNA damage checkpoint signaling | 1.89E-08 | CDKN1A;CCNB1;TFDP1;PCNA;CDK2;E2F1;CDK1;EP300;AURKA;FBXO31 |
|  | GO 2021 | DNA damage response, signal transduction by p53 class mediator resulting in cell cycle arrest | 1.42E-06 | CDKN1A;CCNB1;TFDP1;PCNA;E2F1;CDK1;EP300;AURKA |
|  | GO 2021 | mitotic DNA damage checkpoint signaling | 2.10E-06 | MDC1;CDKN1A;FZR1;PLK1;CDK2;CDK1;CLSPN;FBXO31 |
|  | GO 2021 | mitotic G2 DNA damage checkpoint signaling | 2.03E-04 | CDKN1A;FZR1;PLK1;CDK1;CLSPN |
|  | GO 2021 | DNA damage response, signal transduction by p53 class mediator | 9.23E-07 | CDKN1A;CCNB1;TFDP1;PAXIP1;PCNA;E2F1;CDK1;EP300;AURKA |
|  | GO 2021 | DNA integrity checkpoint signaling | 4.10E-04 | CDT1;E2F1;CDC5L;CLSPN;CDC6 |
| immune response | GO 2021 | granulocyte differentiation | 4.15E-04 | CDKN1A;CDK2;E2F1;EP300 |
|  | GO 2021 | regulation of myeloid cell differentiation | 5.40E-04 | HSPA9;MOV10;CREBBP;HDAC1;WDR5;EP300 |
| Other | GO 2021 | ciliary basal body-plasma membrane docking | 5.27E-08 | TUBA1A;PPP2R1A;TUBB;PLK1;CDK1;NEK2;TUBG1;TUBB4B;NINL;TUBA4A;HAUS1 |
|  | GO 2021 | cilium assembly | 5.92E-04 | TUBA1A;PPP2R1A;TUBB;PLK1;CDK1;NEK2;TUBG1;TUBB4B;NINL;TUBA4A;HDAC6;HAUS1 |
|  | GO 2021 | axo-dendritic transport | 2.26E-04 | APP;HSPA8;APBA1 |
|  | KEGG 2021 | Gap junction | 2.38E-04 | TUBA1C;TUBA1A;TUBB2A;TUBB;CDK1;TUBB4B;TUBA4A |
|  | KEGG 2021 | Cellular senescence | 4.99E-06 | CCNA2;CCNA1;CCNB1;CDKN1A;TGFB1;MYC;CDK2;CDK1;E2F1;BTRC;CDC25A |
|  | GO 2021 | positive regulation of transferase activity | 2.99E-05 | CCT2;TGFB1;PCNA;MYC;PLK1;TCP1;NEK2;AURKB;FGFR1;CCT4 |
|  | GO 2021 | RNA localization to Cajal body | 1.29E-04 | CCT2;TCP1;CCT4 |
|  | GO 2021 | scaRNA localization to Cajal body | 1.29E-04 | CCT2;TCP1;CCT4 |
|  | GO 2021 | cellular response to UV | 1.35E-04 | CDKN1A;CREBBP;PCNA;MYC;EP300;CDC25A;AURKB |
|  | GO 2021 | regulation of cellular response to heat | 1.56E-04 | HSPA8;CREBBP;HSPA1L;HSF1;EP300;NUP98;HSPA1A |
|  | GO 2021 | regulation of cellular response to stress | 2.36E-04 | HSPA8;CREBBP;HSPA1L;HSF1;EP300;NUP98;MCL1;HSPA1A |
|  | GO 2021 | cellular response to light stimulus | 6.19E-04 | CREBBP;PCNA;MYC;EP300;CDC25A;AURKB |
|  | GO 2021 | cellular response to hypoxia | 4.56E-04 | CREBBP;EGLN3;UBB;MYC;UBC;EP300;HIF1AN;SIRT2 |
|  | GO 2021 | error-prone translesion synthesis | 4.98E-04 | MAD2L2;PCNA;UBB;UBC |
|  | GO 2021 | cellular response to light stimulus | 6.19E-04 | CREBBP;PCNA;MYC;EP300;CDC25A;AURKB |
|  | GO 2021 | regulation of mRNA stability | 8.87E-04 | HSPA8;XPO1;UBB;UBC;E2F1;YBX1;AXIN2;HSPA1A |
| Protein localizazion | GO 2021 | positive regulation of establishment of protein localization to telomere | 2.03E-13 | CCT3;CCT6A;CCT2;TCP1;CCT8;CCT7;CCT5;CCT4 |
|  | GO 2021 | regulation of establishment of protein localization to telomere | 6.01E-13 | CCT3;CCT6A;CCT2;TCP1;CCT8;CCT7;CCT5;CCT4 |
|  | GO 2021 | regulation of protein localization to Cajal body | 6.01E-13 | CCT3;CCT6A;CCT2;TCP1;CCT8;CCT7;CCT5;CCT4 |
|  | GO 2021 | positive regulation of protein localization to Cajal body | 6.01E-13 | CCT3;CCT6A;CCT2;TCP1;CCT8;CCT7;CCT5;CCT4 |
|  | GO 2021 | positive regulation of protein localization to chromosome, telomeric region | 1.56E-12 | CCT3;CCT6A;CCT2;TCP1;CCT8;CCT7;CCT5;CCT4 |
|  | GO 2021 | positive regulation of protein localization to nucleus | 3.91E-12 | CCT3;YAP1;CCT2;TGFB1;PLK1;CCT6A;TCP1;UBR5;CDK1;CCT8;CCT7;CCT5;CCT4 |
|  | GO 2021 | positive regulation of telomerase RNA localization to Cajal body | 1.75E-11 | CCT3;CCT6A;CCT2;TCP1;CCT8;CCT7;CCT5;CCT4 |
|  | GO 2021 | positive regulation of establishment of protein localization | 1.62E-10 | CCT3;CCT6A;CCT2;TCP1;CCT8;CCT7;CCT5;CCT4 |
|  | GO 2021 | positive regulation of establishment of protein localization to mitochondrion | 2.15E-04 | TFDP1;HSPA1L;E2F1;HUWE1;TP63;TP73 |
|  | GO 2021 | regulation of protein-containing complex assembly | 2.19E-04 | CDT1;HSPA8;TGFB1;HSPA5;HSF1;RPS3;EP300;HDAC6 |
|  | GO 2021 | positive regulation of cellular protein localization | 6.62E-04 | YAP1;CDT1;TGFB1;DDRGK1;PLK1;CDK1 |
|  | GO 2021 | regulation of protein localization to nucleus | 8.88E-04 | YAP1;TGFB1;PLK1;CDK1;OTUD7B |
|  | GO 2021 | positive regulation of intracellular protein transport | 9.45E-04 | TGFB1;TFDP1;HSPA1L;UBR5;E2F1;HUWE1;TP63;TP73 |
| Protein modification and degradation | GO 2021 | proteasome-mediated ubiquitin-dependent protein catabolic process | 1.45E-35 | ANAPC15;RNF34;ANAPC16;CUL3;CCNF;CUL1;BUB1B;RNF4;ANAPC10;AURKB;ANAPC11;AURKA;CDC20;CCNB1;FZR1;CDC23;PTTG1;UBB;CDC26;CDC27;UBC;SPOP;FBXO5;BUB3;SKP2;BTRC;FBXW5;ANAPC7;HSPA5;UBE2C;PLK1;HUWE1;SIRT2;FBXO31;UBE2S;HECW2;CDC16;CDK2;CDK1;ANAPC4;ANAPC5;ANAPC1;ANAPC2;MAD2L1 |
|  | GO 2021 | modification-dependent protein catabolic process | 6.91E-25 | ANAPC15;RNF34;ANAPC16;CUL3;BUB1B;ANAPC10;AURKB;ANAPC11;AURKA;CDC20;FZR1;CDC23;PTTG1;UBB;CDC26;CDC27;UBC;BUB3;BTRC;ANAPC7;UBE2C;PLK1;CHFR;UBE2S;CDC16;ANAPC4;ANAPC5;ANAPC1;ANAPC2;MAD2L1 |
|  | GO 2021 | ubiquitin-dependent protein catabolic process | 2.75E-24 | ANAPC15;RNF34;ANAPC16;CUL3;BUB1B;OTUD7B;RNF4;ANAPC10;AURKB;ANAPC11;AURKA;CDC20;FZR1;CDC23;PTTG1;CDC26;CDC27;SPOP;BUB3;SKP2;BTRC;FBXW5;ANAPC7;UBE2C;PLK1;HUWE1;SIRT2;CHFR;UBE2S;HECW2;CDC16;ANAPC4;ANAPC5;ANAPC1;ANAPC2;MAD2L1 |
|  | GO 2021 | protein K11-linked ubiquitination | 2.71E-23 | ANAPC13;ANAPC7;UBE2C;RNF4;ANAPC10;ANAPC11;FZR1;CDC23;UBE2S;CDC26;CDC27;CDC16;ANAPC4;ANAPC5;ANAPC1;ANAPC2 |
|  | KEGG 2021 | Ubiquitin mediated proteolysis | 6.34E-22 | ANAPC13;ANAPC7;UBE2C;CUL3;CUL1;HUWE1;ANAPC10;ANAPC11;CDC20;FZR1;CDC23;UBB;UBE2S;CDC26;CDC27;UBC;UBR5;CDC16;ANAPC4;ANAPC5;BTRC;SKP2;ANAPC1;ANAPC2 |
|  | GO 2021 | protein polyubiquitination | 2.15E-20 | ANAPC13;RNF34;CUL3;CCNF;CUL1;RNF4;ANAPC10;ANAPC11;FZR1;CDC23;UBB;CDC26;CDC27;UBR5;UBC;SPOP;SKP2;BTRC;FBXW5;ANAPC7;UBE2C;HUWE1;CHFR;FBXO31;UBE2S;HECW2;CDC16;ANAPC4;ANAPC5;ANAPC1;ANAPC2 |
|  | GO 2021 | regulation of ubiquitin protein ligase activity | 1.30E-13 | CDC20;MAD2L2;FZR1;UBE2C;UBE2S;RPL23;PLK1;FBXO5;BTRC;MAD2L1 |
|  | GO 2021 | protein ubiquitination | 8.28E-12 | PCNA;RNF34;ANAPC16;CUL3;CCNF;CUL1;RNF4;ANAPC11;CDC23;UBB;CDC27;UBR5;UBC;SPOP;SKP2;BTRC;FBXW5;ANAPC7;UBE2C;USP22;PLK1;HUWE1;CHFR;FBXO31;UBE2S;HECW2;CDC16;TRIM33 |
|  | GO 2021 | protein modification by small protein conjugation | 5.92E-11 | FBXW5;PCNA;RNF34;ANAPC7;ANAPC16;UBE2C;CUL3;CCNF;PLK1;CUL1;HUWE1;CHFR;ANAPC11;CDC23;UBB;DDRGK1;UBE2S;HECW2;CDC27;UBC;CDC16;NUP98;BTRC;TRIM33 |
|  | GO 2021 | cellular response to unfolded protein | 4.16E-09 | HSPA9;HSPA8;DAXX;HSPA1L;HSPA5;HSF1;HSPD1;HSPA1A |
|  | GO 2021 | cellular response to topologically incorrect protein | 1.74E-08 | HSPA9;HSPA8;DAXX;HSPA1L;HSPA5;HSF1;HDAC6;HSPA1A |
|  | GO 2021 | positive regulation of ubiquitin protein ligase activity | 1.98E-08 | CDC20;FZR1;UBE2C;UBE2S;PLK1;BTRC |
|  | GO 2021 | protein deubiquitination | 2.75E-08 | USP37;TRRAP;USP22;OTUD7B;CDC25A;CDC20;CCNA2;CCNA1;COPS5;UBB;TNIP2;MYC;UBC;CDK1;EP300;CLSPN;SKP2 |
|  | GO 2021 | histone modification | 3.06E-08 | CCNA2;CREBBP;HDAC2;TRRAP;HDAC1;USP22;CDK2;HUWE1;EP300;SIRT2;AURKB;HDAC6 |
|  | GO 2021 | protein modification by small protein removal | 4.40E-08 | USP37;TRRAP;USP22;OTUD7B;CDC25A;CDC20;CCNA2;CCNA1;COPS5;UBB;TNIP2;MYC;UBC;CDK1;EP300;CLSPN;SKP2 |
|  | GO 2021 | protein stabilization | 5.80E-08 | CCT3;CCT2;CDKN1A;RPL23;HSPD1;CCT6A;TNIP2;TCP1;EP300;CCT8;CCT7;CCT5;CCT4;HSPA1A |
|  | GO 2021 | response to unfolded protein | 5.16E-07 | HSPA9;HSPA8;DAXX;HSPA1L;HSPA5;HSF1;HSPD1;HSPA1A |
|  | GO 2021 | cellular protein modification process | 3.73E-06 | APP;CUL3;CCNF;CUL1;AURKB;AURKA;PPP2CA;PPP2CB;TBK1;PPP2R1A;PPP2R5E;PIM1;WDR5;NEK2;SKP2;BTRC;EGLN3;FBXW5;TGFB1;PLK1;PPP2R5A;SIRT2;CDC25B;FBXO31;COPS5;UBE2S;CDK2;CDK1;FGFR1 |
|  | GO 2021 | 'de novo' posttranslational protein folding | 8.83E-06 | HSPA9;CCT2;HSPA8;HSPA1L;HSPA5;HSPA1A |
|  | GO 2021 | positive regulation of ubiquitin-protein transferase activity | 8.83E-06 | CDC20;FZR1;UBE2C;UBE2S;PLK1;BTRC |
|  | GO 2021 | negative regulation of ubiquitin protein ligase activity | 1.82E-05 | MAD2L2;RPL23;FBXO5;MAD2L1 |
|  | GO 2021 | chaperone cofactor-dependent protein refolding | 7.07E-05 | HSPA9;HSPA8;HSPA1L;HSPA5;HSPA1A |
|  | GO 2021 | negative regulation of ubiquitin-protein transferase activity | 1.50E-04 | MAD2L2;RPL23;FBXO5;MAD2L1 |
|  | GO 2021 | protein-containing complex assembly | 1.56E-04 | YAP1;CREBBP;SAMM50;TRRAP;PPP2R1A;HDAC1;MYC;CUL3;HSF1;CUL1;EP300;AXIN2 |
|  | GO 2021 | regulation of protein serine/threonine kinase activity | 1.67E-04 | CCNA2;PPP2CA;CCNA1;CCNB1;CDKN1A;CCNF;CDC6;CDC25A |
|  | GO 2021 | regulation of proteolysis | 1.78E-04 | HDAC2;IST1;PLK1;SPOP;BTRC;HDAC6 |
|  | GO 2021 | protein destabilization | 2.26E-04 | CREBBP;CUL3;PLK1;EP300;BTRC |
|  | GO 2021 | protein K48-linked ubiquitination | 2.26E-04 | RNF34;UBE2C;CUL3;UBR5;SKP2;RNF4 |
|  | GO 2021 | proteasomal protein catabolic process | 3.70E-04 | FBXW5;RNF34;HECW2;CUL3;SPOP;HUWE1;SKP2;BTRC;RNF4;SIRT2 |
|  | GO 2021 | positive regulation of peptidyl-serine phosphorylation | 3.87E-04 | MAD2L2;APP;TBK1;HAX1;TGFB1;SH2D3C;HDAC6 |
|  | GO 2021 | regulation of peptidyl-serine phosphorylation | 5.08E-04 | MAD2L2;APP;TBK1;TGFB1;HAX1;SH2D3C;HDAC6 |
|  | GO 2021 | regulation of protein catabolic process | 6.22E-04 | MAD2L2;FZR1;HECW2;HUWE1;SIRT2;BANP;MAD2L1 |
|  | GO 2021 | positive regulation of protein phosphorylation | 6.85E-04 | APP;DAXX;CDKN1A;TGFB1;PLK1;SH2D3C;TTK;AXIN2;HDAC6;MAD2L2;TBK1;HAX1;DDRGK1 |
|  | GO 2021 | regulation of protein ubiquitination | 9.07E-04 | DAXX;PAXIP1;UBB;CUL3;RPS3;HUWE1;HSPA1A |
|  | GO 2021 | protein phosphorylation | 9.45E-04 | APP;TGFB1;PLK1;TTK;AURKB;CDC25B;AURKA;CCNA2;TBK1;CDK2;PIM1;CDK1;NEK2;CLSPN;FGFR1 |
|  | GO 2021 | histone H4 deacetylation | 9.82E-04 | HDAC2;HDAC1;SIRT2 |
|  | GO 2021 | modification-dependent macromolecule catabolic process | 9.82E-04 | UBB;UBC;CHFR |
| Signaling | GO 2021 | regulation of signal transduction by p53 class mediator | 1.01E-07 | DAXX;HDAC2;RNF34;HDAC1;RPL23;AURKB;AURKA;CDK2;CDK1;EP300;TP63;BANP;TP73 |
|  | KEGG 2021 | TGF-beta signaling pathway | 5.06E-07 | PPP2CA;PPP2CB;CREBBP;TFDP1;TGFB1;PPP2R1A;MYC;ID1;CUL1;EP300 |
|  | GO 2021 | beta-catenin-TCF complex assembly | 6.09E-06 | CREBBP;HDAC1;TRRAP;MYC;EP300;AXIN2 |
|  | KEGG 2021 | Hippo signaling pathway | 6.45E-06 | YAP1;PPP2CA;PPP2CB;RASSF1;TGFB1;PPP2R1A;MYC;ID1;AXIN2;BTRC;TP73 |
|  | GO 2021 | stress-activated protein kinase signaling cascade | 8.44E-05 | UBB;TNIP2;UBC;CUL1;BTRC |
|  | KEGG 2021 | FoxO signaling pathway | 3.48E-04 | CCNB1;CREBBP;CDKN1A;TGFB1;PLK1;CDK2;EP300;SKP2 |
|  | GO 2021 | stress-activated MAPK cascade | 3.55E-04 | UBB;TNIP2;SH2D3C;UBC;CUL1;BTRC |
|  | GO 2021 | regulation of transforming growth factor beta receptor signaling pathway | 5.59E-04 | CREBBP;TGFB1;UBB;UBC;EP300;TRIM33;HSPA1A |
| Telomeres | GO 2021 | positive regulation of telomere maintenance via telomerase | 1.80E-11 | CCT3;CCT6A;CCT2;TCP1;NEK2;CCT8;CCT7;AURKB;CCT5;CCT4 |
|  | GO 2021 | positive regulation of telomere maintenance via telomere lengthening | 4.58E-11 | CCT3;CCT6A;CCT2;TCP1;NEK2;CCT8;CCT7;AURKB;CCT5;CCT4 |
|  | GO 2021 | regulation of telomerase RNA localization to Cajal body | 9.68E-11 | CCT3;CCT6A;CCT2;TCP1;CCT8;CCT7;CCT5;CCT4 |
|  | GO 2021 | regulation of telomere maintenance via telomerase | 2.11E-09 | CCT3;CCT6A;CCT2;TCP1;NEK2;CCT8;CCT7;AURKB;CCT5;CCT4 |
|  | GO 2021 | positive regulation of telomerase activity | 1.48E-05 | CCT2;MYC;TCP1;NEK2;AURKB;CCT4 |
|  | GO 2021 | regulation of telomerase activity | 7.98E-05 | CCT2;MYC;TCP1;NEK2;AURKB;CCT4 |
| Transcription | GO 2021 | positive regulation of transcription by RNA polymerase II | 1.17E-06 | YAP1;APP;HDAC2;PAXIP1;HDAC1;HOXD13;YBX1;RNF4;HHEX;TBK1;UBB;DDRGK1;MYC;HSF2;UBC;HSF1;E2F1;EP300;TP63;CREBBP;TGFB1;CDC5L;SIRT2;COPS5;TFDP1;HAX1;TNIP2;TP73 |
|  | GO 2021 | positive regulation of transcription, DNA-templated | 6.80E-06 | YAP1;APP;HDAC2;HDAC1;HOXD13;YBX1;RNF4;PHF8;HHEX;TBK1;UBB;DDRGK1;MYC;HSF2;UBC;HSF1;E2F1;EP300;BTRC;TP63;CREBBP;TGFB1;USP22;CDC5L;SIRT2;MAD2L2;COPS5;TFDP1;HAX1;TNIP2;TP73 |
|  | GO 2021 | negative regulation of transcription, DNA-templated | 8.60E-06 | YAP1;HDAC2;HDAC1;GMNN;OTUD7B;AURKB;HDAC6;HHEX;UBB;MYC;UBC;HSF1;MXI1;E2F1;EP300;BTRC;TP63;HSPA8;DAXX;CREBBP;TGFB1;RPL23;PLK1;SIRT2;MAD2L2;ID1;TRIM33 |
|  | GO 2021 | negative regulation of transcription by RNA polymerase II | 4.79E-05 | YAP1;CREBBP;HDAC2;HDAC1;RPL23;PLK1;OTUD7B;SIRT2;AURKB;MAD2L2;HHEX;UBB;MYC;UBC;ID1;HSF1;MXI1;E2F1;EP300;TRIM33;HSPA1A |
|  | GO 2021 | regulation of transcription from RNA polymerase II promoter in response to hypoxia | 1.19E-04 | CREBBP;EGLN3;UBB;UBC;EP300;HIF1AN;SIRT2 |
|  | GO 2021 | regulation of transcription by RNA polymerase II | 1.50E-04 | YAP1;APP;KDM5B;CDKN1A;HDAC2;HDAC1;OTUD7B;HOXD13;YBX1;RNF4;AURKB;PHF8;HSPD1;CCNB1;HHEX;TBK1;UBB;DDRGK1;MYC;HSF2;UBC;HSF1;MXI1;E2F1;EP300;TP63;CREBBP;TGFB1;RPL23;PLK1;CDC5L;SIRT2;MAD2L2;MOV10;COPS5;TFDP1;HAX1;TNIP2;ID1;CDK1;TRIM33;TP73 |
|  | GO 2021 | regulation of gene expression | 1.98E-04 | YAP1;APP;TRRAP;HDAC1;HOXD13;YBX1;HDAC6;AURKA;PPP2CA;PPP2R1A;DDRGK1;MYC;UBR5;RPS3;E2F1;EP300;DAXX;CREBBP;TGFB1;RPL23;MOV10;CILP;HNRNPF;CDK2;TP73;HSPA1A |
|  | GO 2021 | regulation of transcription from RNA polymerase II promoter in response to stress | 2.60E-04 | CREBBP;EGLN3;UBB;UBC;EP300;HIF1AN;HSPA1A |
|  | GO 2021 | regulation of transcription, DNA-templated | 4.15E-04 | YAP1;KDM5B;CDKN1A;HDAC2;PCNA;TRRAP;HDAC1;GMNN;HOXD13;YBX1;RNF4;HDAC6;PHF8;HSPD1;PPP2CA;HHEX;PPP2R1A;MYC;HSF2;HSF1;MXI1;E2F1;EP300;FBXO5;BTRC;TP63;HSPA8;CDT1;DAXX;CREBBP;TGFB1;USP22;CDC5L;CDC6;SIRT2;MAD2L2;CCNA1;TFDP1;ID1;TRIM33;TP73 |
|  | GO 2021 | positive regulation of nucleic acid-templated transcription | 4.19E-04 | YAP1;CREBBP;HDAC2;TGFB1;HDAC1;USP22;RNF4;PHF8;MAD2L2;HHEX;MYC;E2F1;EP300;BTRC;TP63;TP73 |
|  | GO 2021 | negative regulation of gene expression | 7.29E-04 | YAP1;APP;MOV10;RNH1;TGFB1;DDRGK1;CILP;HDAC1;RPS3;YBX1;AURKA;HSPA1A |
|  | GO 2021 | negative regulation of transcription from RNA polymerase II promoter in response to stress | 9.82E-04 | HIF1AN;SIRT2;HSPA1A |
